# Urine-normetanephrine, a predictor of mortality risk in patients with adrenal adenomas

**DOI:** 10.1038/s41598-025-94951-w

**Published:** 2025-04-01

**Authors:** Albin Kjellbom, Magnus Löndahl, Malin Danielsson, Henrik Olsen, Ola Lindgren

**Affiliations:** 1 Department of Clinical Sciences, Faculty of Medicine, Lund University, Lund, Sweden; 2 Department of Endocrinology, Central Hospital Kristianstad, J A Hedlunds Väg 5, 291 33 Kristianstad, Sweden; 3 Department of Endocrinology, Skåne University Hospital, Lund, Sweden; 4 Department of Endocrinology, Ängelholm Hospital, Ängelholm, Sweden

**Keywords:** Adrenal adenoma, Adrenal incidentaloma, Catecholamines, Metanephrines, Endocrine system and metabolic diseases, Adrenal gland diseases

## Abstract

Urine-metanephrines are used in the screening for pheochromocytomas in patients with adrenal incidentalomas, but their potential as markers for mortality in patients with adrenal adenomas has not been studied. A retrospective cohort study was designed to investigate if urine-metanephrines were associated with mortality in patients with adrenal adenomas. Participants where consecutively included between 2005 and 2015 at two endocrine centres in southern Sweden and followed until December 31st, 2022. The exposures were 24 h-urine (tU) metanephrine and normetanephrine analysed at inclusion. The endpoint was all-cause mortality. Outcome data were obtained from the Cause of Death Register. 879 adult (≥ 18 years) patients with an incidentally discovered adrenal adenoma were included in the study and followed for a median of 9.9 years. Median age of patients was 66.7 years, and 59.6% were women. 278 patients died during follow-up. tU-normetanephrine was associated with increased mortality, adjusted hazard ratio (HR) 1.47 (95% CI, 1.27–1.69) (HR for an increase of 100 μmol/mol creatinine). There was no significant association between tU-metanephrine and mortality, HR 0.96 (0.64–1.43). tU-normetanephrine seems to be a predictor for mortality in patients with adrenal adenomas. This widely available diagnostic test may be helpful in further risk-stratifying patients with adrenal adenomas.

## Introduction

Adrenal incidentalomas are common and most often consist of an adrenal adenoma, in which the most frequent endocrine disruption is mild autonomous cortisol secretion (MACS)^1^. Several studies have shown MACS to be associated with increased mortality risk^2–6^. Our understanding of the mechanisms driving this increased risk remains limited, making the identification of high-risk patients a persistent clinical challenge.

Catecholamines are known to play a role in several states of disease, the most obvious being catecholamine-producing tumours, pheochromocytoma, and paraganglioma, but alterations in the sympathetic nervous system (SNS) activity have also been implicated, in for example, heart failure, obstructive sleep apnea and chronic obstructive pulmonary disease^7–9^. Recently, urine levels of the catecholamine metabolites, metanephrines (normetanephrine and metanephrine), have been associated with hypertensive cardiomyopathy, metabolic syndrome, and microalbuminuria in patients investigated for secondary hypertension or adrenal incidentaloma^10,11^. Urine-metanephrines are widely used in the work-up of patients with adrenal incidentalomas because of their excellent sensitivity in screening for pheochromocytoma, but their potential as a marker for mortality risk in patients with adrenal adenomas has not been explored^1^.

We aimed to investigate if urine-metanephrines are associated with mortality risk in a cohort of patients with adrenal adenomas discovered as incidentalomas.

## Methods

### Study design

We conducted a retrospective cohort study at two centres in southern Sweden. The study was approved by the regional ethical review board in Lund, Sweden. Due to the retrospective nature of the study the regional ethical review board in Lund, Sweden, waived the need of obtaining informed consent. All methods were performed in accordance with the Declaration of Helsinki, and the study is reported in accordance with the STROBE guidelines.

### Setting

The endocrine referral centres at two regional hospitals in southern Sweden constituted the study sites (Helsingborg Hospital and Skåne University Hospital). Sweden has a universal health care system; thus, if in need of endocrine health care, the adult inhabitants in the present catchment areas would have been referred to one of the study sites. No competing endocrine centres were available during the study period. Since 2005, regional guidelines have recommended that all patients with adrenal incidentaloma be referred to their regional endocrine clinic for evaluation. Patients referred to the study sites between January 1st, 2005, and August 15th, 2015, because of an adrenal incidentaloma were studied. Patients were evaluated clinically, biochemically and by radiology. They underwent a 1-mg overnight dexamethasone suppression test (DST). A post-test cortisol < 50 nmol/L was considered normal, while a value ≥ 50 nmol/L could indicate MACS^1^. Biochemical screening for pheochromocytoma was done with either 24-h urine (tU) or plasma metanephrines. In the case of hypertension or hypokalaemia, screening for primary aldosteronism was performed. If the tumour was not classified as a lipid-rich adenoma on initial imaging, a minimum of one additional examination with unenhanced computer tomography was conducted after six months. Recommendation on surgical intervention was made after individual assessment by a multidisciplinary team at Skåne University Hospital.

### Participants

All adult (≥ 18 years) patients referred to the study sites because of a previously unknown adrenal incidentaloma during the study period were enrolled. Exclusion criteria were: adrenal incidentaloma < 10 mm, non-adenoma lesion (cysts, haemorrhage, myelolipoma etc.), no tU-metanephrines, no DST, metastatic malignancy, pheochromocytoma, primary aldosteronism, clinical Cushing’s syndrome, use of systemic glucocorticoids, medication affecting dexamethasone metabolism, systemic oestrogen treatment, medication affecting metanephrines (Sotalol, tricyclic antidepressants, MAO inhibitors, venlafaxine, and phenoxybenzamine)^12^, and eGFR < 15 ml/min^13^.

Patients with tU-or plasma metanephrines above the upper reference level were evaluated individually based on clinical, biochemical, and radiological findings in accordance with applicable guidelines.

### Procedures

tU-metanephrines were analysed using High-Performance Liquid Chromatography (HPLC) with electrochemical detection until February 28th, 2010, and from March 1st, 2010, Liquid Chromatography-Mass Spectrometry (LC–MS/MS) was used. The reference ranges for metanephrine and normetanephrine were 20–200 μmol/mol creatinine and 80–360 μmol/mol creatinine, respectively^14^. The detection level was 0.1 μmol/L, and the CV 5% at 3.5 μmol/L and 9.0 μmol/L for metanephrine and normetanephrine, respectively. Patients collected 24-h urine in acid-containing containers. In patients with two or more urine samples, results from the first were included in the study. A 1-step competitive immunoassay (Cobas, Roche Diagnostics) was used to analyse plasma cortisol. The reference range was 171 to 536 nmol/L, the CV 2.1% at 94.9 nmol/L, and the detection limit 0.5 nmol/L.

Clinical and biometrical data were collected using the electronic medical record from the patient’s first visit to the study site. Hypertension was defined as treated arterial hypertension and smoker as a current smoker at the first visit. Adrenal incidentaloma size was defined as maximal axial diameter. Computer tomography and magnetic resonance imaging were used for imaging, and assessments were made by clinical radiologists. Data collection, confirmation of the radiological findings, and examination of out-of-range values were conducted by the authors. Reduced renal function was defined as an estimated glomerular filtration rate < 60 ml/min/1.73 m^2^^15^.

Patients were grouped in tertiles based on the results of tU-metanephrine and tU-normetanephrine.

Conversion of tU-metanephrine to μg/g creatinine divide by 0.574, tU-normetanephrine divide by 0.617. To convert cortisol to μg/dL divide by 27.588.

### Outcomes

The endpoint was all-cause mortality. Outcome data were obtained from the National Board of Health and Welfare Cause of Death Register.

### Variables

Predicting variables in this study were tU-metanephrine and tU-normetanephrine. Predefined covariables were age, sex, smoking, diabetes mellitus, previous cardiovascular disease, history of cancer, reduced renal function, treatment with inhaled glucocorticoids, arterial hypertension, heart failure, and cortisol after DST (cortisol_DST_).

### Outcome period

The start of the study was the day of the DST, and the end-of-study was the first of the following: adrenalectomy, emigration, death, or December 31st, 2022.

### Statistical analysis

Multivariable linear regression was used to analyse the correlation between the dependent variables tU-metanephrine/-normetanephrine and the independent variables: age, sex, adenoma size, cortisol_DST,_ and smoking.

Kaplan–Meier plots with Log-rank tests were used to visualize cumulative all-cause mortality in patients grouped according to tertiles based on tU-metanephrine and tU-normetanephrine.

All-cause mortality was analysed using multivariable Cox proportional hazards regression. Two models were used, one including tU-metanephrine and tU-normetanephrine as continuous variables and one comparing mortality risk between patients in the different tertiles of tU-metanephrine and tU-normetanephrine, respectively. Both models included all the predefined covariables and all reported hazard ratios (HR) are adjusted. Cortisol_DST_ was ln-transformed when used in the Cox model due to non-normal distribution. The proportional hazards assumption was tested with Schoenfeld residuals and Martingale residuals. Interaction analysis was performed by expanding the model with a two-way interaction term. Likelihood displacement values vs. follow-up time were used to identify potential outliers of influence.

The functional form of the association between tU-normetanephrine and HR for all-cause mortality was analysed using restricted cubic splines and Wald test for linearity. Differences in baseline characteristics between patient groups were analysed, categorical data were analysed using the Chi-square test, and continuous data using the Mann–Whitney U test. A two-tailed P-value < 0.05 was considered statistically significant. Continuous data were reported as median and interquartile range (IQR), and categorical data as numbers and percentages. Statistical analysis was performed using StataCorp. 2024. Stata Statistical Software: Release 18. College Station, TX: StataCorp LLC.

Sensitivity analysis was performed using E-values, indicating the strength of association an unmeasured confounder would need to nullify the observed association^16,17^.

## Results

**Fig. 1:**
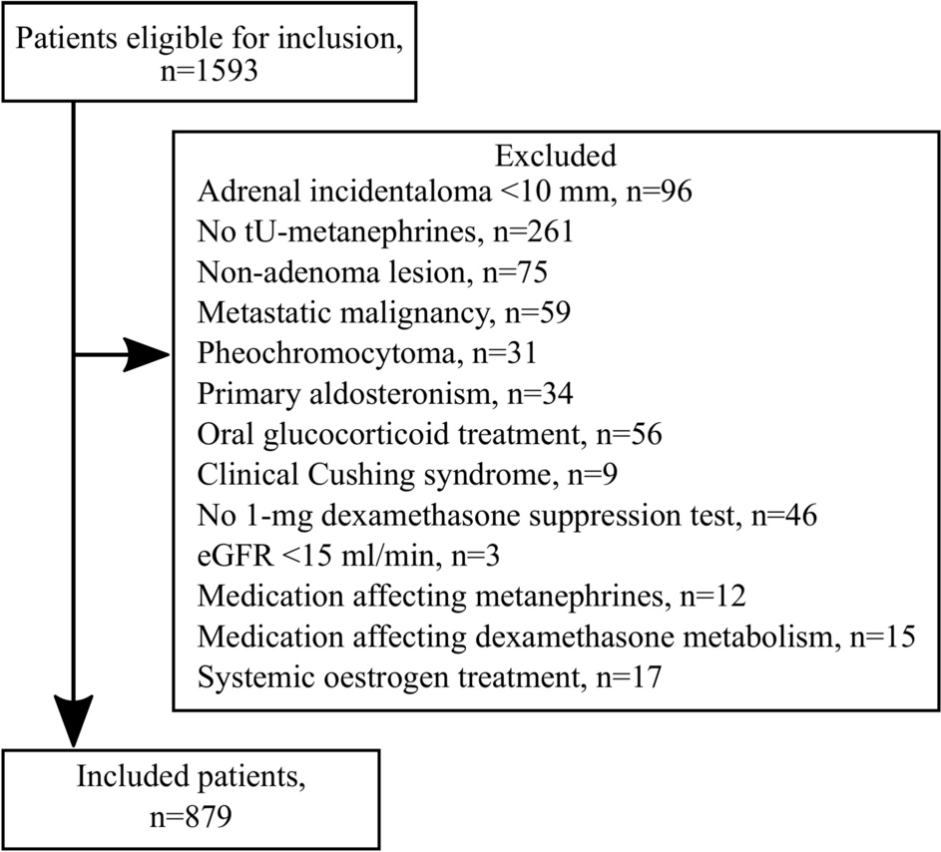
Inclusion flow chart.

Eight hundred seventy-nine patients, 59.6% women, were included during the study period. (Fig. 1) Their median age was 66.7 (58.8–73.5) years, and the observation time was 9.9 (7.9–12.6) years. Baseline characteristics for all patients, grouped in tertiles based on tU-metanephrine and tU-normetanephrine, are shown in Table 1. Tertile 1 contains the lowest values and tertile 3 the highest. The median tU-metanephrine was 59 (43–81) μmol/mol creatinine in women and 55 (41–71) in men. For tU-normetanephrine, the corresponding numbers were 193 (152–248) and 144 (115–184) μmol/mol creatinine, respectively. Pheochromocytoma/paraganglioma had been ruled out in the four patients with tU-metanephrine and the 25 patients with tU-normetanephrine above the upper reference range.

**Table 1:** Characteristics of the study population.

	All	tU-normetanephrine				tU-metanephrine			
	n = 879	Tertile 1 (T1) n = 294	Tertile 2 (T2) n = 294	Tertile 3 (T3) n = 291	p T1/T2 T1/T3 T2/T3	Tertile 1 (T1) n = 295	Tertile 2 (T2) n = 292	Tertile 3 (T3) n = 292	p T1/T2 T1/T3 T2/T3
Women	524 (59.6%)	111 (37.8%)	186 (63.3%)	227 (78.0%)	< 0.001 < 0.001 < 0.001	170 (57.6%)	158 (54.1%)	196 (67.1%)	0.39 0.02 0.001
Age (years)	66.7 (58.8–73.5)	63.5 (53.7–69.9)	66.3 (58.5–72.8)	70.9 (62.3–76.7)	< 0.001 < 0.001 < 0.001	66.4 (56.9–73.0)	65.1 (58.0–71.2)	68.9 (61.1–76.0)	0.51 < 0.001 < 0.001
BMI (kg/m^2^) (n = 857)	27.3 (24.2–31.5)	27.2 (24.7–30.4) (279)	27.7 (24.8–32.1) (288)	26.9 (23.2–31.6) (290)	0.23 0.18 0.03	29.5(26.2–33.6) (286)	27.5 (24.5–31.6) (287)	25.4 (22.4–28.7) (284)	< 0.001 < 0.001 < 0.001
Smoker	318 (36.2%)	82 (27.9%)	102 (34.7%)	134 (46.1%)	0.08 < 0.001 0.005	71 (24.1%)	110 (37.7%)	137 (46.9%)	< 0.001 < 0.001 0.02
Previous cardiovascular disease	188 (21.4%)	57 (19.4%)	58 (19.7%)	73 (25.1%)	0.92 0.10 0.12	62 (21.0%)	60 (20.6%)	66 (22.6%)	0.89 0.64 0.55
Diabetes mellitus	158 (18.0%)	47 (16.0%)	52 (17.7%)	59 (20.3%)	0.58 0.18 0.43	70 (23.7%)	40 (13.7%)	48 (16.4%)	0.002 0.03 0.36
Hypertension	460 (52.3%)	122 (41.5%)	164 (55.8%)	174 (59.8%)	0.001 < 0.001 0.33	164 (55.6%)	142 (48.6%)	154 (52.7%)	0.09 0.49 0.32
Heart failure	39 (4.4%)	12 (4.1%)	8 (2.7%)	19 (6.5%)	0.36 0.19 0.03	17 (5.8%)	6 (2.1%)	16 (5.5%)	0.02 0.88 0.03
Previous cancer	159 (18.1%)	47 (16.0%)	46 (15.7%)	66 (22.7%)	0.91 0.04 0.03	52 (17.6%)	48 (16.4%)	59 (20.2%)	0.70 0.43 0.24
eGFR < 60 ml/min	123 (14.0%)	32 (10.9%)	38 (12.9%)	53 (18.2%)	0.45 0.01 0.09	52 (17.6%)	40 (13.7%)	31 (10.6%)	0.19 0.02 0.25
Inhaled glucocorticoids	87 (9.9%)	21 (7.1%)	22 (7.5%)	44 (15.1%)	0.87 0.002 0.004	26 (8.1%)	30 (10.3%)	31 (10.6%)	0.55 0.46 0.89
Adenoma size (mm)	20.0 (15–26)	19 (15–25)	20 (15–27)	20 (15–26)	0.07 0.08 1.0	20 (15–25)	20 (15–26)	20 (15–27)	0.86 0.29 0.45
Bilateral adenomas	134 (15.2%)	41 14.0%)	47 (16.0%)	46 (15.8%)	0.49 0.53 0.95	38 (12.9%)	44 (15.1%)	52 (17.8%)	0.45 0.10 0.37
tU-metanephrine (μmol/mol creatinine)	57 (42–77)	48 (38–63)	56 (42–72)	71 (54–97)	< 0.001 < 0.001 < 0.001	38 (30–42)	57 (53–64)	88 (77–107)	-
tU-normetanephrine (μmol/mol creatinine)	173 (132–224)	118 (100–131)	173 (158–188)	254 (223–297)	–	148 (117–189)	168 (132–214)	210 (164–262)	< 0.001 < 0.001 < 0.001
Cortisol_DST_ (nmol/L)	47.0 (33–69)	41 (29–58)	47 (33–67)	54 (38–83)	0.001 < 0.001 < 0.001	41 (30–58)	46 (32–65)	55 (39–86)	0.01 < 0.001 < 0.001
Cortisol_DST_ ≥ 50 nmol/L	398 (45.3%)	100 (34.0%)	131 (44.6%)	167 (57.4%)	0.009 < 0.001 0.002	105 (35.6%)	126 (43.2%)	167 (57.2%)	0.06 < 0.001 0.001
All-cause mortality	278 (31.6%)	63 (21.4%)	81 (27.6%)	134 (46.1%)	0.08 < 0.001 < 0.001	87 (29.5%)	78 (26.7%)	113 (38.7%)	0.45 0.02 0.002
Adrenalectomy	42 (4.8%)	15 (5.1%)	15 (5.1%)	12 (4.1%)	1.0 0.57 0.57	14 (4.8%)	16 (5.5%)	12 (4.1%)	0.69 0.71 0.44
Observation time (years)	9.9 (7.9–12.6)	10.1 (8.3–12.5)	10.0 (8.4–12.9)	9.2 (6.7–12.2)	0.97 0.001 0.002	10.3 (8.3–12.1)	10.3 (8.3–13.1)	9.0 (7.3–12.1)	0.19 0.005 < 0.001

Data are median (IQR), or n (%). Differences between groups were analysed using the Chi-square test or the Mann–Whitney test, as appropriate. Conversion of tU-metanephrine to μg/g creatinine divide by 0.574, tU-normetanephrine divide by 0.617. Conversion of cortisol to μg/dL divide by 27.588.

*BMI* body mass index, *eGFR* estimated glomerular filtration.

Multivariable linear regression showed that both tU-metanephrine and tU-normetanephrine had a significant positive correlation with age, female sex, smoking, and cortisol_DST_. Neither tU-metanephrine nor tU-normetanephrine correlated significantly with adenoma size. (Table 2).

**Table 2:** Correlation between tU-metanephrine/normetanephrine and baseline characteristics, analysed with multivariable regression.

	tU-normetanephrine			tU-metanephrine		
	Coefficient (CI 95%)	p	R^2^	Coefficient (CI 95%)	p	R^2^
Age (years)	2.16 (1.70–2.61)	< 0.001	0.22	0.51 (0.32–0.70)	< 0.001	0.12
Woman	50.32 (40.53–60.12)	< 0.001		5.71 (1.62–9.80)	0.006	
Adenoma size (mm)	0.26 (-0.31–0.83)	0.37		-0.13 (-0.37–0.11)	0.28	
Smoking	19.75 (9.38–30.12)	< 0.001		14.22 (9.89–18.55)	< 0.001	
Cortisol_DST_ (nmol/L)	0.18 (0.09–0.27)	< 0.001		0.11 (0.07–0.15)	< 0.001	

### All-cause mortality

Two hundred seventy-eight patients died during the study period. The number of deaths in each tertile based on tU-metanephrine was 87, 78, and 113, respectively. The corresponding numbers based on tU-normetanephrine tertiles were 63, 81, and 134.

**Fig. 2:**
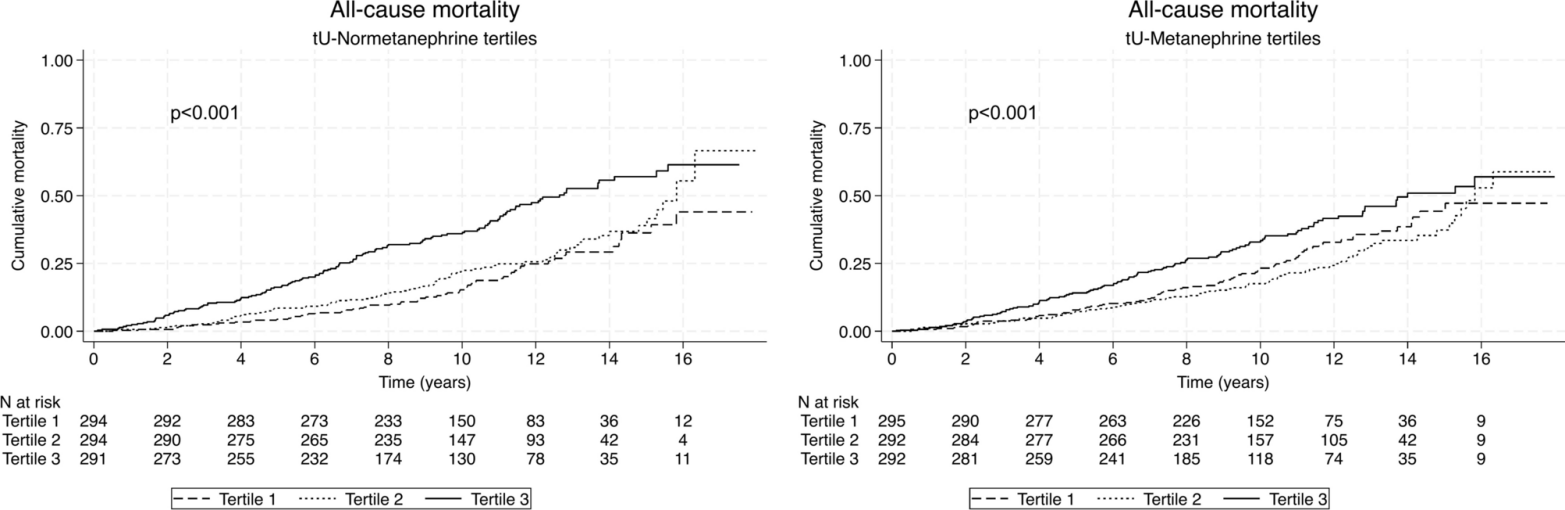
All-cause mortality. Kaplan–Meier plots comparing cumulative mortality between patients grouped in tertiles based on tU-normetanephrine, and tU-metanephrine, respectively. P-values were calculated using the log-rank test.

Log-rank test showed a significant difference in all-cause mortality between patients grouped in tertiles according to tU-metanephrine and normetanephrine, with a higher mortality in the upper tertiles. (Fig. 2).

Multivariable Cox proportional hazards regression including tU-metanephrine and tU-normetanephrine as continuous variables (results given for increase by 100 μmol/mol creatinine) alongside the other predefined covariables showed a significant increase in mortality risk associated with tU-normetanephrine, HR 1.47 (1.27–1.69), while no significant association was observed for tU-metanephrine, HR 0.96 (0.64–1.43).

Patients in tertile 3 of tU-normetanephrine had significantly increased mortality risk compared with patients in tertile 1 and 2, HR 1.84 (1.25–2.70) and 1.49 (1.09–2.03), respectively. There was no significant difference in mortality risk between tertile 2 and 1, HR 1.15 (0.80–1.65). Excluding the patients with tU-normetanephrine above the upper reference range did not significantly change the results. Analysing patients grouped according to tU-metanephrine tertiles showed a lower mortality risk in tertile 2 compared to tertile 1, HR 0.67 (0.48–0.94). (Table 3) There was no violation of the proportional hazards assumption, and Martingale residuals supported the inclusion of the variables tU-metanephrine and tU-normetanephrine untransformed.

**Table 3:** Adjusted hazard ratios for all-cause mortality.

	tU-Normetanephrine				tU-Metanephrine			
	Continuous variable	Tertile 2 vs. 1	Tertile 3 vs 2	Tertile 3 vs. 1	Continuous variable	Tertile 2 vs. 1	Tertile 3 vs 2	Tertile 3 vs. 1
All-cause mortality, HR (95% CI)	1.47 (1.27–1.69)	1.15 (0.80–1.65)	1.49 (1.09–2.03)	1.84 (1.25–2.70)	0.96 (0.64–1.43)	0.67 (0.48–0.94)	1.16 (0.85–1.60)	0.81 (0.59–1.13)

Hazard ratio (HR) adjusted for age, sex, smoking, diabetes mellitus, previous cardiovascular disease, history of cancer, reduced renal function, treatment with inhaled glucocorticoids, hypertension, heart failure, cortisol_DST_, and tU-metanephrine/normetanephrine. HR for continuous variables is given for an increase of 100 μmol/mol creatinine.

### Sub-group analysis

Interaction analysis revealed a significant interaction between tU-normetanephrine and smoking (p = 0.043). Analysis of the association between mortality and tU-normetanephrine, as a continuous variable, in smokers and non-smokers separately showed a significant association in both groups, with HR 1.76 (1.37–2.26) in smokers and 1.35 (1.12–1.63) in non-smokers.

### Functional form of association

**Fig. 3:**
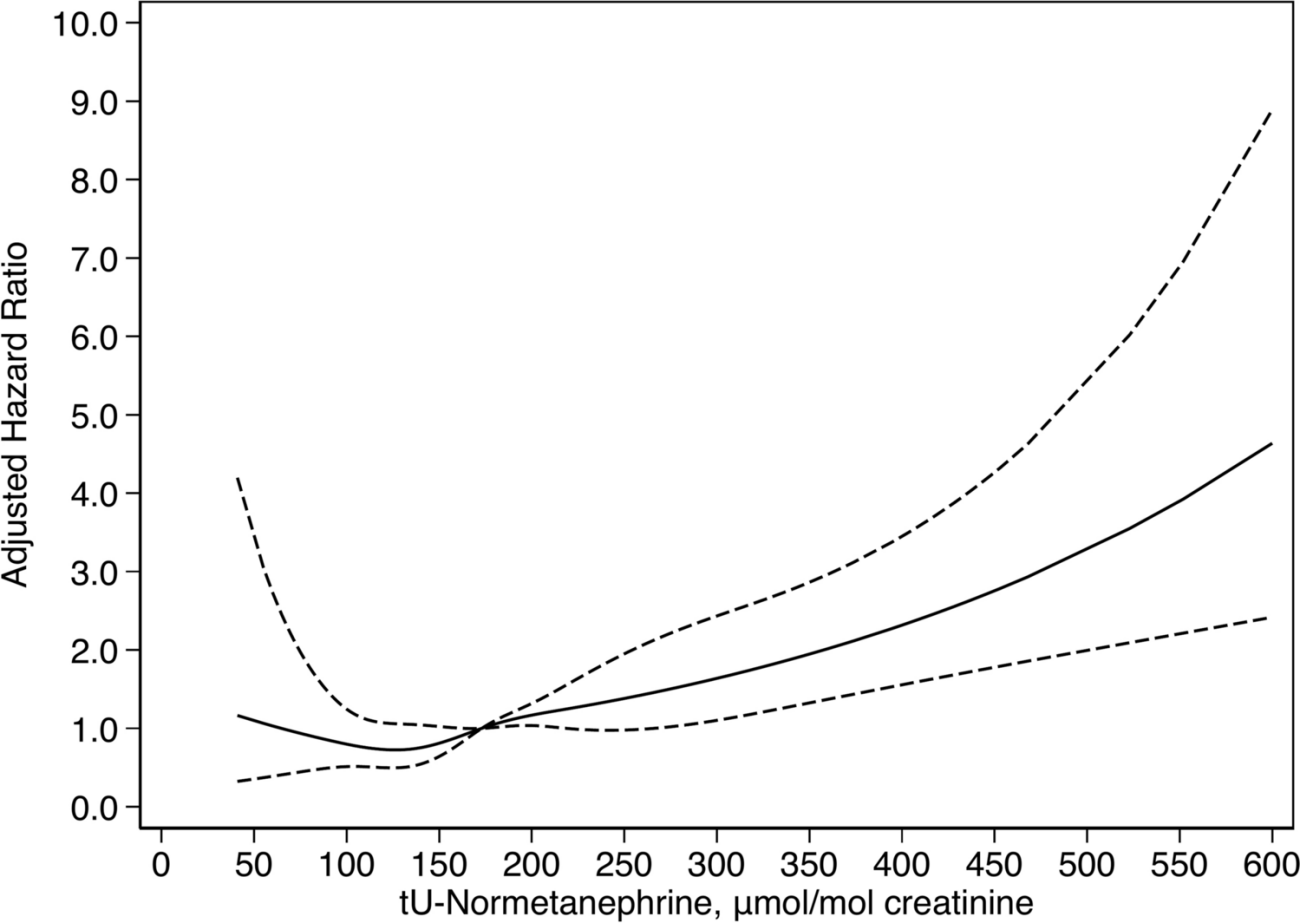
Functional form of the association between tU-normetanephrine and all-cause mortality. Adjusted hazard ratio 1.0 set at population median of tU-normetanephrine (173 μmol/mol creatinine). Wald test for linearity not opposing linearity (p = 0.46).

The functional form of the association between tU-normetanephrine and all-cause mortality was analysed using restricted cubic splines. Figure 3 shows the functional form of the association between tU-normetanephrine and adjusted HR (95% CI) for all-cause mortality. Wald test for linearity did not oppose linearity (p = 0.46).

### Sensitivity analysis

E-values for the HR and the 95% CI for all-cause mortality in patients of tU-normetanephrine tertile 3 compared with tertile 1, were 2.42 and 1.61. E-values for the HR and the 95% CI for all-cause mortality associated with tU-normetanephrine, analysed as a continuous variable, were 1.94 and 1.64.

## Discussion

The results of this study suggest that tU-normetanephrine is an independent, clinically relevant predictor for mortality risk in people with adrenal adenomas. The results may also indicate a link between autonomous cortisol secretion and the SNS.

Catecholamine function and metabolism are complex. While the vast majority of metanephrine measured in urine is of adrenal origin, the case of normetanephrine is more complicated^18^. tU-normetanephrine measures both conjugated and free normetanephrine, with the former constituting more than 95% of the measured normetanephrine in urine. Conjugated normetanephrine is foremost produced in mesenteric organs and primarily reflects the norepinephrine production of these organs, which accounts for approximately 50% of all norepinephrine produced in the body. The rest originates from spill-over from other parts of the SNS and the adrenal glands^19^. At rest, the excretion of epinephrine is low, but in response to stressors such as physiological stress, e.g. hypoglycaemia, it increase more than norepinephrine. Norepinephrine, on the other hand, has a constant, varying tone, reflecting the activity of the SNS^20^. Even though there are more refined methods of measuring SNS activity, such as microneurography, normetanephrine in urine can be viewed as a marker of SNS tone^7,19,21^.

An increased sympathetic tone is associated with various disease states, not limited to catecholamine-producing tumours. One of the most robust associations is that between heart failure and increased SNS activity^8,22,23^. Catecholamine levels have also been associated with adverse outcomes in persons with type-2 diabetes and in elderly^24,25^. Chronic obstructive pulmonary disease likewise seems to be associated with increased SNS activity^9^. In contrast, the results concerning hypertension, and at least SNS activity measured by urine catecholamines, are more conflicting^21^. In this study we found that tU-normetanephrine was associated with an increased mortality risk in people with adrenal adenomas also after adjusting for co-morbidities. This indicates that tU-normetanephrine is an independent risk factor and suggests that there may be additional risk-driving factors caused by, or causing, increased normetanephrine production in addition to the known co-morbidities. Interestingly, the association between mortality risk and tU-normetanephrine appeared linear, with validity also within the reference range. Another important observation in our cohort was the increased rate of women in the higher tertiles of normetanephrine. This aligns with previous findings showing higher normetanephrine levels in women^7^. However, there was no indication that the risk associated with tU-normetanephrine differed between women and men. Results from the subgroup analyses of smokers suggest that smoking and SNS activity may have a synergistic detrimental effect. However, data on former smoking status and smoking duration were unavailable, limiting our ability to further investigate this potential relationship. The signal of a possible association between tU-metanephrine and mortality was weak, and the results might imply a U-shaped association, although discrete.

The mortality rate in the studied population was high. Previous research has shown that patients with adrenal incidentalomas have a higher mortality rate than the general population^26,27^. While this can be partly explained by a higher prevalence of cardiovascular comorbidities and risk factors such as smoking^26,28,29^, the high prevalence of MACS in patients with adrenal incidentalomas appears to play a crucial role in the increased mortality risk^2–6,27^.

Due to our study´s design, we cannot determine if the levels of metanephrines are directly affected by the adrenal adenoma. However, the observed positive correlation between cortisol_DST_ and metanephrines suggests a possible link between adrenal adenomas and the SNS, or more precisely, between autonomous cortisol secretion and SNS tone. A bidirectional interaction between the adrenal medulla and cortex is well established^30,31^. Numerous animal models have shown that glucocorticoids increase synthesis and release of catecholamines with subsequent effects on the cardiovascular system^32–34^. This could imply that cortisol secretion has a stimulatory effect on the SNS and provides a possible mechanistic explanation behind the increased mortality risk seen in patients with MACS. On the other hand, the reverse interaction is also possible with catecholamines stimulating cortisol secretion^31,35,36^.

tU-metanephrines is an established screening tool for pheochromocytomas in patients with adrenal incidentalomas. Our findings suggest that tU-normetanephrine, additionally, might prove a clinically helpful marker for risk assessment in patients with adrenal incidentalomas constituted by adenomas. So far, the dexamethasone suppression test is the cornerstone in the evaluation, diagnosis, and risk stratification of patients with MACS^1^. Possibly, tU-normetanephrine may prove useful in further risk-stratifying patients with MACS, for example, when considering a patient for adrenalectomy. However, being the first study investigating an association between tU-metanephrines and mortality in patients with adrenal adenomas the results need to be validated by additional studies, and a possible interaction between adenoma cortisol secretion and the SNS further explored. A question of interest would be to investigate tU-metanephrines before and after surgery in patients with adrenal adenomas with and without MACS.

We recognize the study has several limitations. A substantial number of patients were excluded based on the predefined exclusion criteria. The largest excluded group consisted of patients lacking tU-metanephrines, the majority of whom were screened using plasma metanephrines. The baseline characteristics of this group were similar to those of the included patients. Although the study was designed to minimize the risk of selection bias, we acknowledge that it cannot be entirely ruled out. As mentioned earlier, the study design only allows conclusions to be drawn concerning associations and not causal relationships, and even though results are adjusted for known confounders and potential sources of error are excluded, there is a risk of unmeasured bias. Such an unmeasured confounder would, however, according to the E-values, have to be considerable to nullify the observed association^17^.

In conclusion, this is the first study to show tU-normetanephrine as a continuous independent predictor of all-cause mortality in patients with adrenal adenomas discovered as incidentalomas.

## Data Availability

Some or all datasets generated during and analysed during the current study are not publicly available but are available from the corresponding author upon reasonable request.
